# Novel Single-Staged Posterior Retropleural Approach with Thoracoscopic Guidance for Resection of a Thoracic Dumbbell Schwannoma

**DOI:** 10.7759/cureus.6548

**Published:** 2020-01-03

**Authors:** Jared Sweeney, Stephanie Zyck, Mark Crye, Michael Galgano

**Affiliations:** 1 Neurosurgery, State University of New York Upstate Medical University, Syracuse, USA; 2 Thoracic Surgery, State University of New York Upstate Medical University, Syracuse, USA

**Keywords:** spine, neoplasms, neurosurgery, spine neurosurgery, tumor resection

## Abstract

Dumbbell spinal cord tumors are infrequent pathologic entities. The optimal approach to safe surgical resection is ill-defined and must often be individualized. This is assisted with multiple tumor classification systems. Here, we describe a novel technique used to safely and successfully resect a large thoracic dumbbell schwannoma originating from the left T3 spinal nerve root with extension into the posterior mediastinum adjacent to the parietal pleura and thoracic aorta. A review of the literature was performed to study described surgical approaches to primary spinal dumbbell tumors. The decision-making process and preoperative imaging for operative planning are included. A detailed description of the procedure follows with intraoperative images. Gross total resection with no neurologic sequelae was achieved. Previously described operative techniques for resection of primary spinal dumbbell tumors with advantages and limitations of each are then reviewed. Gross total resection was safely achieved utilizing a single-staged posterior retropleural approach with anterior thoracoscopic guidance. The tumor was removed en bloc through a large posterior window. The prone position was utilized for the entire case with no intraoperative repositioning required. No intraoperative or immediate postoperative complications occurred. We report a novel approach to resecting a large primary spinal dumbbell tumor. A single-stage retropleural approach with anterior thoracoscopic guidance facilitated safe and successful gross total resection. Maintenance of the prone position throughout surgery allowed for reduced operative time, excellent anterior, and posterior visualization and no added patient morbidity. Repositioning to the lateral decubitus position may not be required in select cases.

## Introduction

Primary spinal tumors (PST) are relatively uncommon, accounting for approximately 15% of central nervous system (CNS) tumors [1]. Of these, “dumbbell-shaped” PST accounts for approximately 18% [2]. Their characteristic name is derived from their unique morphology, consisting of intraspinal and extraspinal tumor components, with tumor segments connected through the intervertebral foramen [3-4]. Most causative pathologies are benign, the most common of which is schwannoma [2]. Most present late with symptoms of cord compression, as extraspinal tumor growth into paraspinal tissues occurs much faster than intraspinal growth limited by small spinal canal size [3-5]. Cervical spine locations are most common (44%), followed by lumbosacral (29%) and thoracic (27%) locations [3]. Surgical intervention is warranted in patients with progressive neurological deficits or radiologic evidence of tumor enlargement [1,3,5]. Incidentally found, asymptomatic tumors may be managed conservatively with regular follow up and imaging [1]. When surgical intervention is indicated, gross total resection (GTR) should be attempted, when safe, to reduce the risk of tumor recurrence [5-6]. The first step in determining the optimal surgical approach is determining the tumor’s size, location, characteristics, and proximity to adjacent structures. Using this information, the surgical approach is then tailored to the individual patient’s pathology. Several tumor classification systems have been developed to assist in this decision making [7-8]. Due to the rarity of dumbbell tumors, the optimal surgical approach to safe resection is controversial. Here, we report our experience with a novel, single-staged, posterior approach utilizing thoracoscopic guidance to safely resect a large T3 schwannoma causing profound thoracic myelopathy and occupying the posterior mediastinum.

## Technical report

**Figure 1:**
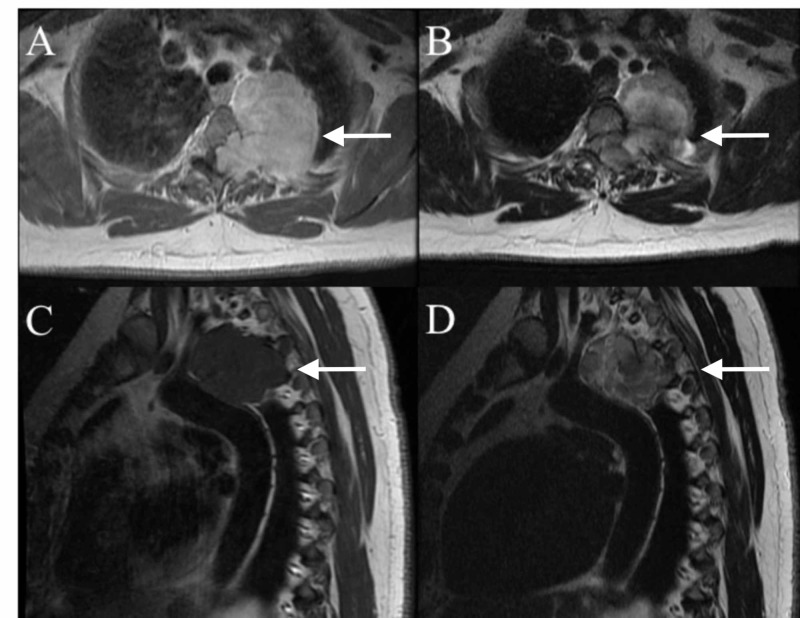
Preoperative MRI thoracic spine with and without contrast Preoperative T1 axial MRI with gadolinium (A) and T2 axial MRI without contrast (B) showing dumbbell schwannoma intraspinal and extraspinal components. Preoperative T1 (C) and T2 (D) sagittal MRI showing dumbbell schwannoma extending into the posterior mediastinum adhering to the thoracic aorta. MRI, magnetic resonance imaging

A 57-year-old woman presented with three months of progressively worsening intrascapular pain extending to the left lower thorax, lower extremity weakness, numbness, and gait disturbance. Thoracic computed tomography (CT) and magnetic resonance imaging (MRI) were obtained demonstrating a large mass originating from the left T3 neural foramen, with extension into the posterior mediastinum and invasion into the entire T3 pedicle, much of the T3 vertebral body, and the ipsilateral T2/3 and T3/4 facet joints (Figure 1A-1D).

**Figure 2:**
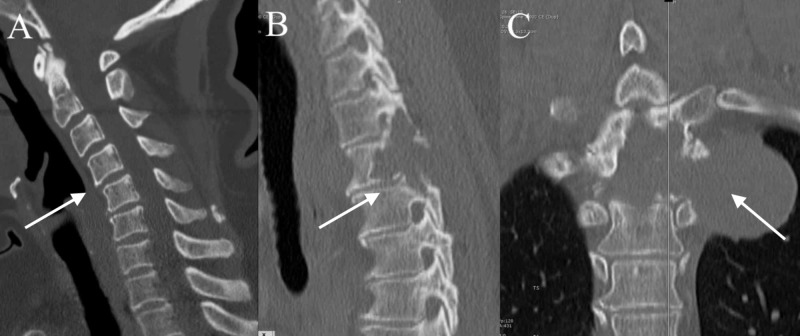
Preoperative CT thorax without contrast Preoperative CT thorax sagittal view without contrast (A) showing cervical kyphosis pronounced at C5. Preoperative CT thorax sagittal view (B) and coronal view (C) demonstrating tumor involvement of the entire T3 pedicle, much of the T3 vertebral body, the ipsilateral T2/3 and T3/4 facet joints, and the superior aspect of the T4 pedicle. CT, computed tomography

There was also invasion into the superior aspect of the T4 pedicle (Figure 2A-2C).

**Figure 3:**
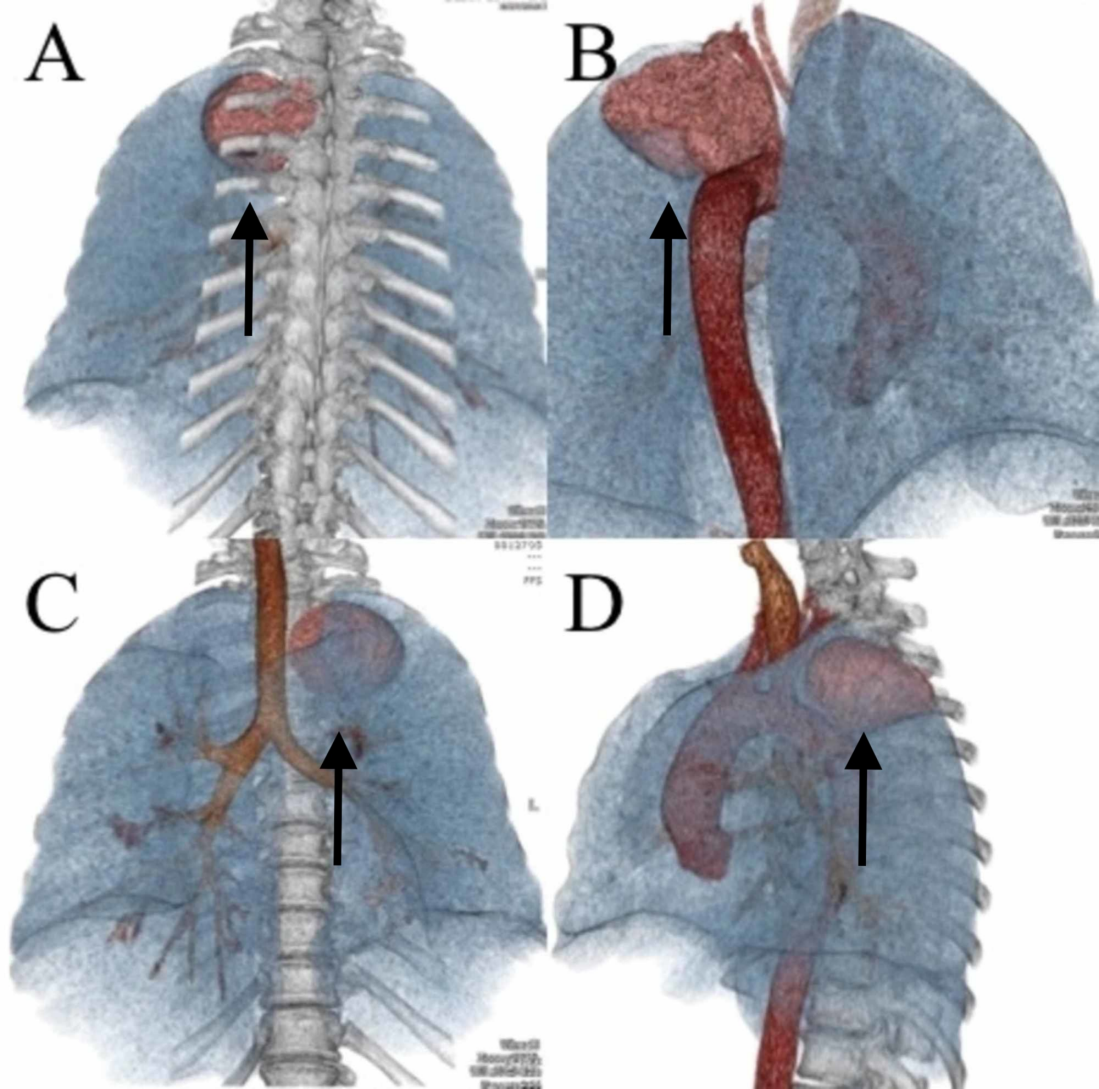
Preoperative 3D CT thorax reconstruction Posterior view (A), posterolateral view with vertebral column removed (B), anterior view (C), and lateral view (D) 3D, three-dimensional; CT, computed tomography

The mass caused profound compression upon the spinal cord. Extraspinal tumor dimensions were 42 mm x 68 mm x 49 mm, and intraspinal tumor dimensions were 10 mm x 10 mm. Preoperative CT core-guided biopsy was consistent with a schwannoma. Surgical options were discussed due to worsening neurological deficit, and the decision was made to proceed with surgical resection. A CT 3D reconstruction of the thorax was obtained for surgical planning (Figure 3A-3D).

**Figure 4:**
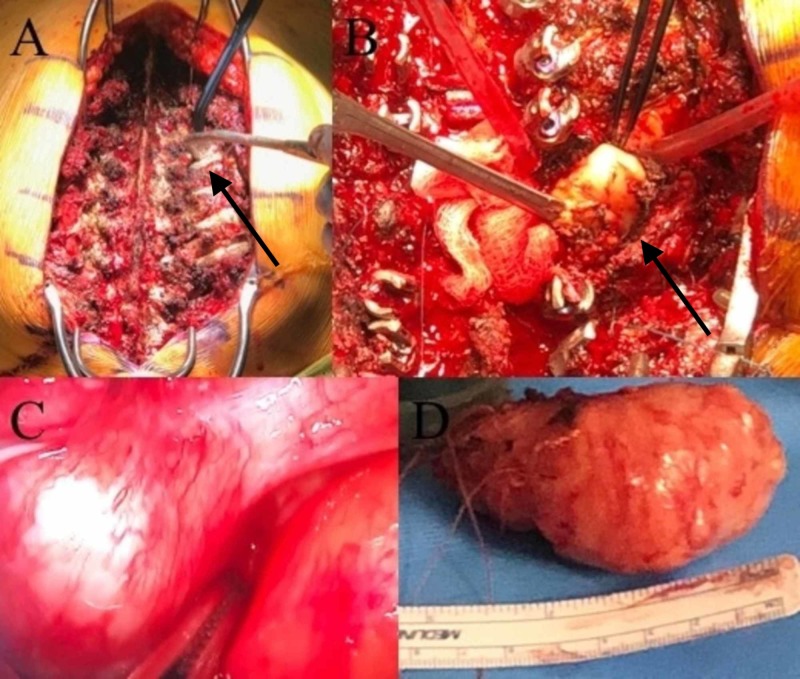
Intraoperative images Intraoperative posterior exposure (A). Dissection and expression of tumor from posterior retropleural approach after removing bilateral T2-4 lamina, left T2-4 facets, and 4cm of the left T2-4 ribs and transverse processes (B). Video-assisted thoracoscopic view of the schwannoma (left) against the aorta (right) within the mediastinum (C). Gross specimen of schwannoma after resection en bloc (D).

The patient was positioned prone and the left hemi-thorax was also included in the operative field in anticipation of thoracoscopy. The patient was prepared and draped in an appropriate fashion to allow both vertebral manipulation and thoracoscopy without changing patient position during the operation. A midline incision was made in the posterior cervical-thoracic region, and standard subperiosteal dissection was undertaken to expose the posterior elements. The left T2-4 ribs were also exposed, as they would need to be taken down in an effort to gain access to the posterior mediastinal component of the tumor (Figure 4A).

We felt that the patient would require supplemental spinal instrumentation due to the anticipation of iatrogenic instability after the tumor resection, in addition to pre-existing cervical kyphosis [3,5].

A laminectomy was then performed from T2-4 to gain access to the intraspinal component of the tumor (Figure 4B). The left-sided T2/3 and T3/4 facet joints were eroded by the tumor. We then found the origin of the tumor attached to the left T3 nerve root. We ligated the nerve root proximal to the dorsal root ganglion, then cauterized and amputated the tumor as it exited the neural foramen. This allowed us to deliver the tumor that was within the spinal canal, neural foramen, and vertebral body of T3 in one piece after carefully dissecting it away from the dura. 

Next, we defined the plane between the undersurface of the ribs and the endothoracic fascia from T2-4 on the left. An ultrasonic bone cutter was utilized to resect approximately 4 cm of the ribs after disarticulating them from the costo-transverse joint. This maneuver allowed us to gain access to the retropleural space (Figure 4B). The posterior margin of the tumor was then identified. Bipolar cautery was used to shrink the tumor. Two 0’vicryl sutures were placed into the substance of the tumor for the purpose of mobilizing it in various directions during the retropleural dissection. Multiple cottonoids were placed within the plane between the pleura and tumor capsule. Once the majority of tumors had been expressed from the posterior mediastinum, we called in the thoracic surgeons to assist with direct visualization of the anterior tumor dissection.

The thoracic surgeons gained intrathoracic access to visualize the tumor from the mediastinum and assist with expression of the tumor posteriorly under direct thoracoscopic guidance (Figure 4C). Notably, the patient remained prone for this portion of the operation. Cursory evaluation of the pleural space showed no evidence of any pleural-based disease other than the known posterior mediastinal mass. From the thoracic space, the ipsilateral lung was temporarily collapsed to improve visualization and blunt dissection with a freer device was undertaken to identify a plane between the pleura and tumor capsule. Utilizing direct thoracoscopic guidance, the mass was freed circumferentially from the pleura and removed posteriorly through the posterior incision (Figure 4D). 

Upon inspection of the subpleural capsule space, there was no evidence of any residual tumor. A 28-French chest tube was placed in the initial port site and guided posteriorly towards the apex. At that point in time, dual lung ventilation was resumed.

Cervical-thoracic instrumentation was then completed after titanium rods were contoured and placed, in addition to allograft. A sub-fascial drain was placed and the site was closed in the usual fashion. 

**Figure 5:**
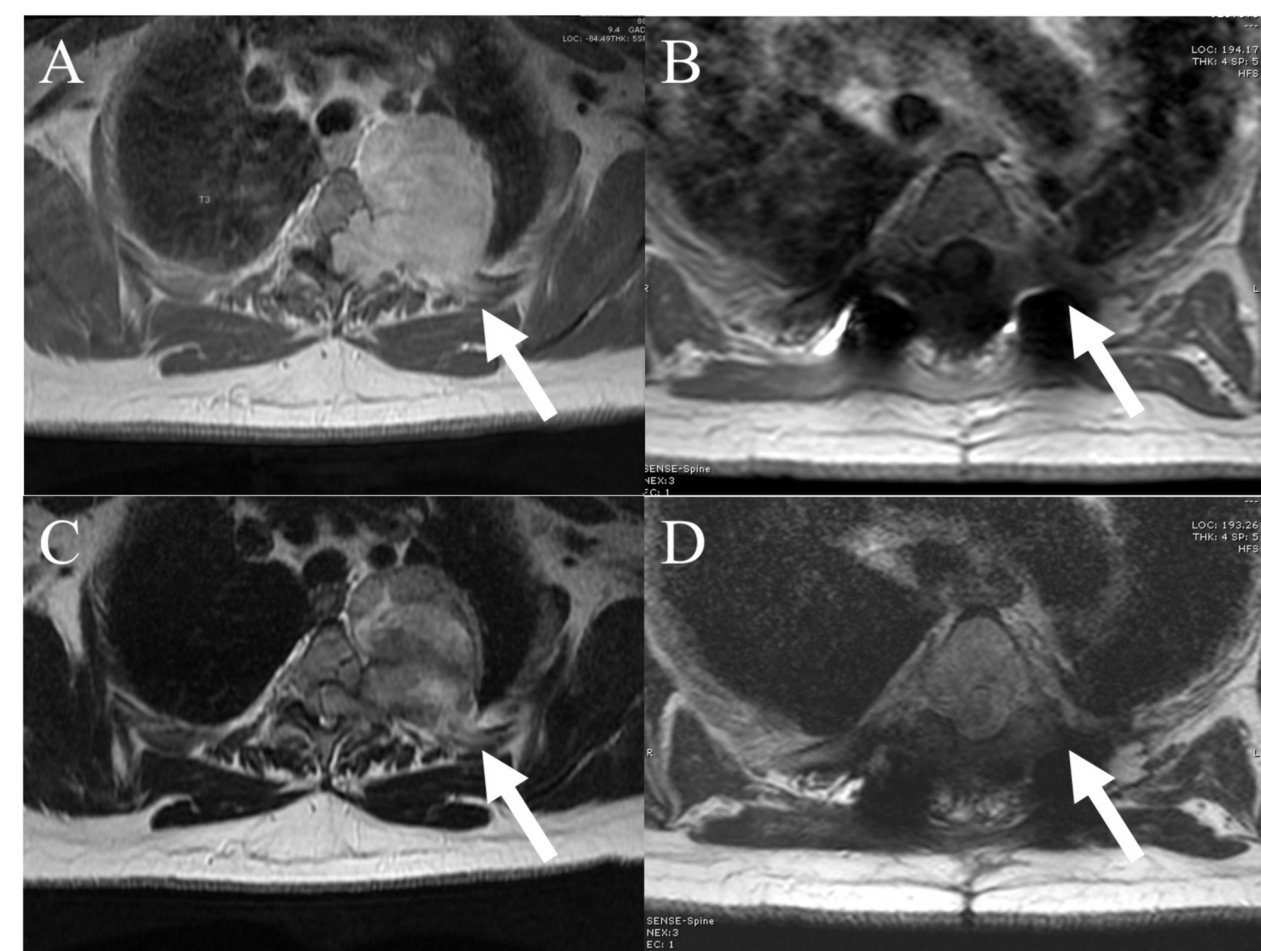
Comparison of preoperative and postoperative MRI thoracic spine with and without contrast Comparison of pre-operative T1 with gadolinium axial MRI (A) with post-operative TI with gadolinium axial MRI (B). Comparison of pre-operative T2 axial MRI (C) with post-operative T2 axial MRI (D). MRI, magnetic resonance imaging

Postoperative imaging revealed complete tumor removal (Figure 5A-5D).

**Figure 6:**
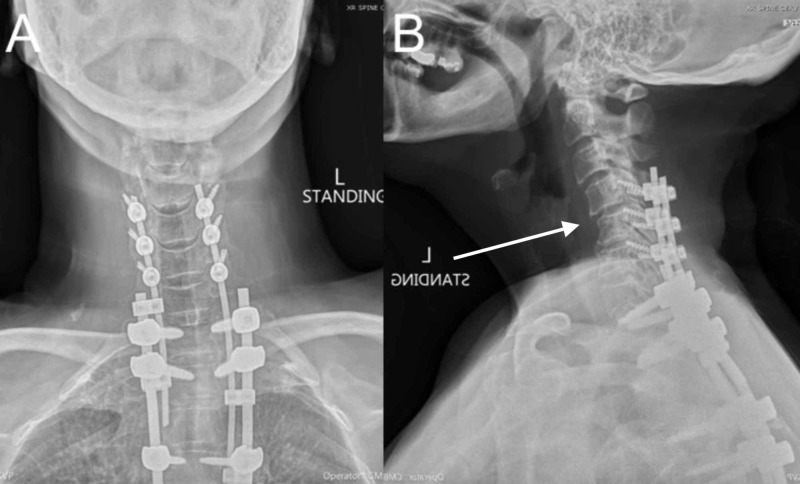
Postoperative cervical spine X-rays Plain cervical spine X-rays, anteroposterior view, showing rostral intact hardware from C4-T7 (A). Plain cervical spine X-rays, lateral view, showing rostral intact hardware from C4-T7 (B).

Optimization of spinal alignment was confirmed (Figure 6).

After several weeks of inpatient rehabilitation, the patient’s gait function began steadily improving. At her 10-month follow-up, imaging has continued to reveal no tumor recurrence, and good alignment in the sagittal plane with continued neurological improvements. 

## Discussion

The surgical approach to thoracic dumbbell tumors is complex and should be individualized [9-10]. Various approaches have been described in the literature including approaches according to the Eden and/or Toyama classification (Tables 1-2) [1,7-10].

**Table 1:** Eden classification of spinal dumbbell tumors based on gross anatomical location Classification by Eden et al. [8]

Type	Tumor location
Type I	Intradural and extradural
Type II	Intradural, extradural, foraminal, and paravertebral
Type III	Extradural, foraminal, and paravertebral
Type IV	Foraminal and paravertebral

**Table 2:** Toyama classification of spinal dumbbell tumors based on MRI shape and three-dimensional location Classification by Asazuma et al. [7] *Subtypes based on the extent of extra-foraminal tumor extension MRI, magnetic resonance imaging

Type	Tumor location	Subtype (if present)*
Type I	Intradural and extradural	n/a
Type II	Extradural with compression in intervertebral foramen	A – Extradural and foraminal
		B – Extradural and paravertebral
		C – Foraminal and paravertebral
Type III	Intradural and extradural with compression in intervertebral foramen	A – Intradural, extradural, foraminal
		B – Intradural, extradural, and paravertebral
Type IV	Extradural and intravertebral (invasion of vertebral body)	n/a
Type V	Extradural and extralaminal (invasion of lamina)	n/a
Type VI	Extradural with multidirectional invasion of bone	n/a

Here, we describe a case of a large schwannoma originating from the left T3 spinal nerve root with extension into the posterior mediastinum adjacent to the parietal pleura and thoracic aorta, with ipsilateral vertebral body, pedicle, and facet joint destruction. This tumor was classified as Eden Type III and Toyama Type IIB. Resection was planned utilizing a single-staged, posterior retropleural approach utilizing anterior thoracoscopic guidance in the prone position. Complete tumor resection was achieved, in addition to optimized spinal alignment. 

It is accepted that a single posterior approach is effective for resecting tumors with a predominantly intraspinal component [1]. A posterior approach should be performed first to decompress the spinal cord and release the tumor from the nerve root. This minimizes spinal cord manipulation and trauma during subsequent tumor removal [5,10]. Tumors with paraspinal extension often warrant an anterior approach that can be invasive, theoretically increasing postoperative morbidity [1]. It is at this stage where disagreement on the optimal management of dumbbell tumors arises, primarily consisting of whether an anterior approach is necessary and if so, what type of anterior approach should be utilized. The following discussion will include alternative management strategies for thoracic dumbbell tumors, with advantages and disadvantages of each described. It is important to note that the optimal approach is chosen based on the individual patient’s tumor characteristics and classification [7-8].

*Single-Stage Posterior-Only Approach* 

A single-stage posterior approach involving laminectomy, constotransversectomy, facetectomy, and sometimes pediculectomy typically ipsilateral to the tumor at has been reported in the literature with success in achieving good surgical outcomes [1,9-11]. Notably, the rate of GTR was 50% in one series by Aldo et al. [10] and 80% in a more recent series by Zairi et al. [1].

Advantages to the single-stage posterior approach may include limited muscle damage, blood loss, operative time, and postoperative pain [11]. Additionally, only one surgical incision is required in contrast to multiple required to perform a combined approach. Disadvantages include blind posterior manipulation during resection that can lead to intrathoracic injury [12]. A single-stage posterior approach is not indicated if there is potential for malignant tumor pathologies suggested by preoperative imaging, as this approach does not allow adequate visualization of tumor margins [10].

Single Stage Anterior Only Approach

Singe-stage anterior-only approaches have been described for Eden type IV dumbbell tumors with significant foraminal and paravertebral extension. Both thoracotomy and video-assisted thoracoscopic surgery (VATS) have been utilized with success [13]. Wang et al. reported successful resection of a schwannoma extending into the middle mediastinum, with extension anterior to the trachea and posterior to the superior vena cava (SVC), causing cough and SVC syndrome. GTR was achieved with VATS alone, with no tumor recurrence or postoperative complications [14].

Single-Stage Combined Posteroanterior Approach with Video-Assisted Thoracoscopic Surgery

Combined posterior laminectomy and VATS has been introduced as an alternative method to remove thoracic dumbbell tumors [1,5,12,13,15]. Open thoracotomy is infrequently used for the resection of dumbbell spinal tumors since the advent of VATS in the 1990s. 

A combined approach offers many advantages for resection of dumbbell tumors as evidenced by our experience. Anterior approaches allow for less posterior bone removal as the extent of tumor visualization required posteriorly is much less [5]. This may reduce spinal instability and the extent of instrumentation required [10]. VATS allows for better visualization of tumor in relation to delicate surrounding mediastinal structures, and better visualization of the tumor-spine interface, in comparison to posterior approaches alone [5]. A disadvantage of using a combined approach with VATS may be the reduced ability to control intraoperative bleeding. Bleeding in the highly vascular posterior or middle mediastinum may obscure the operative field. VATS enables only limited bleeding control, increasing the risk of atelectasis or pleural effusion [9-10,15]. In our experience, VATS allowed for adequate visualization and avoidance of delicate vasculature to avoid bleeding complications. An additional disadvantage is that anterior approaches often require tube thoracostomy placement, increasing the risk of postoperative pain, pulmonary dysfunction, and infections [9-10].

Similar success has been reported in the literature utilizing a combined approach with VATS. Konno et al. reported a series of three dumbbell tumors and two paraspinal tumors of the thoracic spine resected with a combined approach, in the cases of dumbbell tumors, or VATS alone in the cases of paraspinal tumors. GTR was achieved in all five patients [13]. Ohya et al. reported a series of two dumbbell schwannomas of the T1 nerve root, one of which was adherent to the left vertebral and subclavian arteries. A combined approach, with VATS performed first to free the tumor from the delicate vasculature, resulted in GTR in both patients [12]. Nam et al. reported a series of seven patients with thoracic dumbbell tumors, including four schwannomas, two neurofibromas, and one hemangioma. All seven cases resulted in GTR with two postoperative complications, including atelectasis and facial anhidrosis from ipsilateral sympathetic trunk injury [15]. Prieto et al. reported a case of a giant thoracic dumbbell schwannoma, measuring intraspinally at 7-mm maximum diameter and extraspinally at 65-mm maximum diameter, with displacement and compression of the spinal cord. GTR was achieved and the patient recovered completely with no complications [5].

Patient Positioning For Combined Posteroanterior Approach With Video-Assisted Thoracoscopic Surgery

Repositioning of patients to the lateral decubitus position may not be required to perform a posteroanterior approach to dumbbell tumor resection. Utilizing a combined posteroanterior approach commonly involves performing a two-step operation where patient positioning must be changed. The posterior approach is often completed with the patient prone, while the anterior VATS is often completed in the lateral decubitus position to allow adequate intrathoracic visualization and exposure of the anterior chest wall [5,15]. Patient repositioning was completed in each of the above case series citing a combined posteroanterior approach and is well described in the literature [5,12-13,15].

After extensive planning with the help of thoracic surgery, we elected to leave the patient prone to both the anterior and posterior steps of tumor resection. To facilitate this, the left upper extremity was not taped down and the field was manipulated to expose the left anterior chest wall, allowing for adequate VATS access to the thorax without changing patient position or requiring preceding posterior closure. Additionally, prone positioning allowed for maintenance of vertebral column alignment through all stages of the operation and minimized opportunity for damage to proximal structures. This helped safely reduce operative time while allowing excellent and convenient visualization of tumor resection from anterior and posterior perspectives. 

## Conclusions

We report a case of large dumbbell schwannoma of the left T3 nerve root with extension into the posterior mediastinum adjacent to the parietal pleura and thoracic aorta (Eden Type III, Toyama Type IIB). A single-stage extended posterior retropleural approach with anterior thoracoscopic guidance was used to achieve a GTR with optimized results. The authors advocate that a single-stage combined approach with posterior tumor dissection and anterior thoracoscopic visualization for improved safety and delicate steps of anterior dissection may be effective to achieve GTR in appropriate patients. Avoiding repositioning by maintaining the prone position may safely shorten operative time, allow excellent visualization of both the anterior and posterior operative fields simultaneously, and add no additional patient morbidity in select cases. 
